# Evolution of health care utilization and expenditure during the year before death in 2015 among people with cancer: French snds-based cohort study

**DOI:** 10.1007/s10198-021-01304-1

**Published:** 2021-06-07

**Authors:** Audrey Tanguy-Melac, Dorian Verboux, Laurence Pestel, Anne Fagot-Campagna, Philippe Tuppin, Christelle Gastaldi-Ménager

**Affiliations:** Caisse Nationale de l’Assurance Maladie (Cnam) , 50 avenue du Professeur André Lemierre, 75020 Paris, France

**Keywords:** End-of-life, Cancer, Healthcare expenditure, France, Out-of-pocket, Administrative database, I10, I14, I18

## Abstract

**Background:**

Cancer patients have one of the highest health care expenditures (HCE) at the end of life. However, the growth of HCE at the end of life remains poorly documented in the literature.

**Objective:**

To describe monthly reimbursed expenditure during the last year of life among cancer patients, by performing detailed analysis according to type of expenditure and the person’s age.

**Method:**

Data were derived from the *Système national des données en santé* (SNDS) [national health data system], which comprises information on ambulatory and hospital care. Analyses focused on general scheme beneficiaries (77% of the French population) treated for cancer who died in 2015.

**Results:**

Average reimbursed expenditure during the last year of life was €34,300 per person in 2015, including €21,100 (62%) for hospital expenditure. "Short-stays hospital" and "rehabilitation units" stays expenditure were €14,700 and €2000, respectively. Monthly expenditure increased regularly towards the end of life, increasing from 12 months before death €2000 to €5200 1 month before death. The highest levels of expenditure did not concern the oldest people, as average reimbursed expenditure was €50,300 for people 18–59 years versus €25,600 for people 80–90 years. Out-of-pocket payments varied only slightly according to age, but increased towards the end of life.

**Conclusion:**

A marked growth of HCE was observed during the last 4 months of life, mainly driven by hospital expenditure, with a more marked growth for younger people.

**Supplementary Information:**

The online version contains supplementary material available at 10.1007/s10198-021-01304-1.

## Introduction

The sustained growth of health care expenditure (HCE) accentuates the pressure placed on governments, health insurance and individual budgets [1]. Ageing of the population is one of the main drivers of this growth [2]. As highlighted by the World Health Organization, the proportion of the world population aged 60 + should almost double between 2015 and 2050, and the number of people aged 80 + should increase almost fourfold over the same period [3]. France is not an exception, as recent demographic estimates show that, by 2070, there will be 76.5 million inhabitants (+ 10.7 million people) and that this growth will be essentially due to an increased number of people aged 65 + (+ 10.4 million) [4].

In their seminal study based on Swiss data, Zweifel et al. (1999) concluded that HCE can be more explained by proximity to death (PTD) (1–2 years before death) than person's age [5]. An equivalent effect were reported in other countries for a panel of health care [2, 6–13]. Even in including 260 morbidities in their estimates, Howdon and Rice (2018) demonstrated an effect of PTD on HCE, although this effect was attenuated when comorbidities were considered [2].

As highlighted by the World Health Organization, cancer is the second leading cause of death in the world in 2018. End-of-life people with cancer have a higher HCE than people with other causes of death [14–18]. A study conducted on French administrative data showed that total expenditure for people who died in 2013, all causes combined, was about €17,600^1^[19]. The medical spending considered were those during the calendar year of death. In France, the average annual expenditure for cancer patients 1 year before death was estimated at €36,589 in 2008 [20]. Moreover, spending concentration around several expenditure items has also been studied. For example, in the United States, hospital care accounts for 44.2% of all expenditure during the last year of life [21]. A recent international comparison of seven countries also showed that people who died from cancer had been frequently hospitalized during the last days of life, regardless health systems or organization of end-of-life care differences from one country to another [22].

Literature concerning the dynamics of end-of-life expenditure remains fairly limited. The majority of studies has reported total expenditure for several time frames before death: the last month, the last 6 months or the last year of life, for example [14, 22, 23]. They thus do not specifically study the month after month dynamics of expenditure during last months of life. Furthermore, most studies focused on only a few expenditure items, most often hospital expenditure. For example, a recent study analyzed the end-of-life expenditure of women with an uterine cancer and they only focused their analyses on hospital expenditure [24]. Another study, based on data derived from a health insurer in The Netherlands, analyzed end-of-life expenditure of people with cancer. Hospital stays represented the leading expenditure, with an average of €12,700 for the last year of life and €3517 for the last 30 days of life [25]. Given the high expenses during the last year of life, the question of out-of-pocket (OOP) payments may arise. The few studies conducted showed high OOP payments for people at the end of life [8, 26–28].

The description and analysis of HCE dynamics during the last year of life is an important issue for health insurance. It thus can know the actual costs of cancer at the end-of-life, the intensification of care and the distribution of the expenses among various HCE items. A recent study emphasized the end-of-life hospital-centered approach in France [29] while many studies showed that patients prefer to die at home [22, 30, 31]. As a consequence, description and analysis of end-of-life HCE may help to improve health insurance resources allocation, especially in a context of increasing cancer incidence, while improving accounting patients’ wishes.

This article is designed to complete the literature in several ways. First, it aims to analyze the pattern and the evolution of HCE during the last 12 months of life for patient receiving cancer treatment before they died in 2015. Second, the SNDS allows us to distinguish between several expenditure items in both hospital and ambulatory settings. Finally, thanks to this administrative database OOP payments and their evolution during the last year of life are studied. To the best of our knowledge, no existing study has analyzed OOP payments from this point of view.

### Data and methods

#### Data source

In France, information concerning the healthcare utilization of the entire French population, i.e. more than 66.6 million people, covered by the various compulsory health insurance schemes, are collected in the *Système national des données de santé* (SNDS) [national health data system] [32]. It collects anonymous, individualized and comprehensive data concerning all reimbursed private hospitals and outpatient healthcare utilization but also prescriptions and procedures reimbursed (e.g. physicians, dentist, nurses, drugs, transports, etc.). Individuals’ information (date of birth, sex, town of residence, etc.) are also available.

All of these data are linked, by using a pseudonymized identifier, to data of the national hospital discharge database (PMSI: *programme de médicalisation des systèmes d*’*information*), concerning public stays: short-stay hospitals (“SSH”), rehabilitation units (“Rehab”), hospital-at-home (“HaH”) and psychiatric hospitals. Residence in skilled nursing homes (SNH) can also be determined. Drugs given during a hospital stay are directly included in the Diagnosis-Related Group (DRG) tariffs. It is therefore not possible to know precisely which drugs were prescribed and their particular costs. In order to support access to innovation in health care institutions, some innovative drugs or medical devices are registered on a list, called the “*liste en sus*” which are billable over and above DRG tariffs in short-stay hospitals (SSH). In a synthetic way, SNDS allows us to have information about ambulatory care expenditure and hospitals stays (both public and private sector).

Although, the SNDS does not include clinical data on the results of physician visits, prescriptions or examinations, it however includes information on the presence of one of 30 long-term diseases (LTD) eligible for 100% reimbursement of HCE, including cancer.

#### Population

The general health scheme fund has developed algorithms^2^ based on SNDS data to identify beneficiaries who are reimbursed for chronic or serious or expensive diseases each year. These algorithms are mainly based on diagnosis in short-stay hospitals, LTD, specific drugs or procedures. Patients under treatment for cancer one year before their death (“active cancer”, hereafter) are thus defined over a 2-years period from SSH (cancer-specific diagnosis, chemotherapy or radiotherapy) and/or new applications for LTD during the last 2 years.

This study encompassed all adults (i.e. aged 18 +) who died in 2015 and who were identified as having an active cancer. The population was restricted to beneficiaries of the national health insurance general scheme, because, at this time, the others schemes did not systematically record explicitly the fact that a person was covered by LTD or the vital status of their beneficiaries. In 2015, the general scheme covered about 77% of the French population.

#### Analysis

All total and reimbursed HCE by national health insurance general scheme for each person with at least one health care reimbursement during the year (whether or not this expenditure is related to cancer) were extracted. Total expenditure encompass all expanses presented for reimbursement. Expenditure items costs were available on a monthly basis for all individuals. The following expenditure items were taken into account in the analyses:

- Ambulatory care expenditure: physicians, dentists, paramedical (physiotherapists, nurses, etc.), laboratory tests, drugs (delivered in the pharmacies), medical devices and related services,^3^ transport;
- Hospital expenditure in SSH (including drugs and medical devices out of DRG tariffs), “Rehab”, “HaH”, psychiatry and outpatient visits and procedures;
- Allowances related to sick leave and disability benefits.

In France, expenditure directly related to LTD (cancer in our case) are totally reimbursed by the general scheme. When treatments are not directly related to cancer, 78% of the expenses are covered by the general scheme, 13% by complementary health insurance (CHI) and the rest directly by the households on average. Out-of-pocket payments included in this study were calculated as the difference between the amount of expenditure presented for reimbursement and the reimbursed amount by national health insurance general scheme. Out-of-pocket payments, therefore, include all co-payments as well as any excess fees billed by health care professionals. In France, 95% of the population has access to complementary health insurance [33] and most of these insurances cover a large proportion of co-payments.

For each person, total annual reimbursed expenditure was calculated as the sum of reimbursed expenditure over the last 12 complete months, from month 12 to month 1, excluding the month of death (month 0), which is an extrapolation, submitted to a specific analysis. Expenditure during the last month of life (month 0) was treated specifically to allow comparison with the expenditure of the other months. It was extrapolated from the observed expenditure for the days on which the person was alive during this last month divided by the number of days alive, multiplied by 30.

All statistical analyses were performed with SAS 9.3 software. The CNAM has been granted permanent access to SNDS data by the *French data protection agency* (CNIL).

## Results

### Descriptive statistics

A total of 125,497 people who died in 2015 with an active cancer were included in the study (Table 1). These people had an average age of 73 ± 13 years and 41% were women. About 18% had lung cancer and 12% had colorectal cancer. 52% of people had cardio-neurovascular disease, 28% a chronic respiratory disease and 21% diabetes. All deceased people had at least one hospitalization (SSH, Rehab or HaH) during their last year of life (including the month of death). Regardless of age, most people (67%) died in hospital.

**Table 1:** Sociodemographic characteristics at the end of life of patient with cancer who died in 2015, according to age

*N*		Age-group					
	Total	18–59	60–69	70–79	80–89	≥ 90	*p*
	125,497	20,574	28,743	29,719	34,678	11,783	
%	100	16.4	22.9	23.7	27.6	9.4	
Women	41.2	43.1	35.3	37.1	44.4	53.2	***
Mean age (years, mean ± SD)	73.0 ± 13.3	51.7 ± 7.1	64.9 ± 2.8	74.7 ± 2.9	84.3 ± 2.8	92.7 ± 2.6	***
Cardiovascular and neurovascular disease	51.6	27.6	42.9	54.3	64.8	69.4	***
Diabetes	21.1	10.0	20.9	26.6	24.8	16.2	***
Mental illness	7.8	11.5	8.8	6.6	6.1	6.2	***
Neurological or degenerative disease	13.1	7.1	6.1	9.9	20.1	28.4	***
Chronic respiratory disease	27.7	25.4	31.2	30.2	26.4	21.0	***
Chronic inflammatory disease	3.6	3.1	3.2	3.9	4.0	3.2	***
Rare diseases	0.8	0.7	0.8	1.0	0.8	0.3	***
HIV/AIDS	0.5	1.6	0.5	0.2	0.1	0.1	***
Chronic dialysis	0.9	0.5	0.8	1.2	1.1	0.5	***
Liver or pancreatic disease	14.0	20.3	18.7	14.7	9.0	4.8	***
Other LTD	8.7	6.8	6.5	7.9	10.6	14.1	***
Place of death							***
SSH	66.9	76.6	74.6	69.9	59.4	45.9	
HaH	4.2	4.7	4.2	4.6	3.8	3.1	
Rehab	8.5	5.6	6.7	8.4	11.0	10.4	
SNH	5.3	0.2	1.0	2.5	8.8	21.4	
Other	15.1	13.0	13.5	14.5	16.9	19.2	
At least one stay during the year and mean length of stay, % (mean number of days ± SD)							
SSH	97.6 (53 ± 45)	99.1 (66 ± 51)	99.0 (60 ± 47)	98.4 (55 ± 46)	96.7 (43 ± 39)	91.9 (31 ± 29)	***
HaH	10.7 (52 ± 72)	15.6 (58 ± 77)	12.1 (54 ± 72)	11.1 (51 ± 70)	8.0 (47 ± 66)	5.4 (47 ± 74)	
Rehab	25.3 (52 ± 54)	16.7 (58 ± 65)	20.1 (53 ± 58)	25.5 (52 ± 55)	32.6 (51 ± 49)	31.5 (49 ± 46)	**
All types	100 (70 ± 66)	100 (84 ± 73)	100 (76 ± 68)	100 (73 ± 67)	100 (62 ± 61)	100 (46 ± 53)	***
SNH	8.8 (174 ± 118)	0.4 (153 ± 114)	1.9 (172 ± 118)	4.6 (164 ± 119)	14.8 (165 ± 118)	33.4 (189 ± 117)	***

Source: SNDS, All of France, General scheme + SLM

*p < 0.05

**p < 0.01

***p < 0.001

Analyses revealed fairly different results according to age. First, 28% of our study’s population was 80–89 years, ahead of the 70–79 years age-group which represented 24% of the study’s population. Inversely, less than 10% of people were aged 90 + . The prevalence of certain cancers also varied considerably according to age group. People who died and who have a lung cancer were younger: 26% were aged 18–59, while only 4.5% were aged 90 + . An opposite trend was observed for prostate cancer: men aged 18–59 represented 1.3% of the population, while men 90 years and older represented 17%. An increasing prevalence of "cardiovascular and neurovascular disease" and "neurological or degenerative disease" was observed with age. Finally, the presence of "mental illness", "HIV/AIDS" and "liver and pancreatic diseases" decreased with increasing age.

The places of death differed according to age, although hospitals remained the leading place of death (67% of deaths) regardless of age. The youngest people mostly died in SSH (77%), while the places of death were more varied for the oldest people: 46% in SSH, 21% in SNH and 10% in Rehab. Almost all people (98%) of the study population, regardless of age, had had at least one SSH stay during the 12 months preceding death. Hospitalization rates varied slightly according to age: 99% for people aged 18–59 to 92% for people aged 90 + . As expected, almost one-third of people aged 90 + had at least one SNH stay versus only 0.4% of people younger than 60. HaH rates tended to decline with increasing age (from 15.6 to 5.4%).

### Total reimbursed expenditure by items and monthly evolution of healthcare utilization rate during the last 12 months of life

**Fig. 1:**
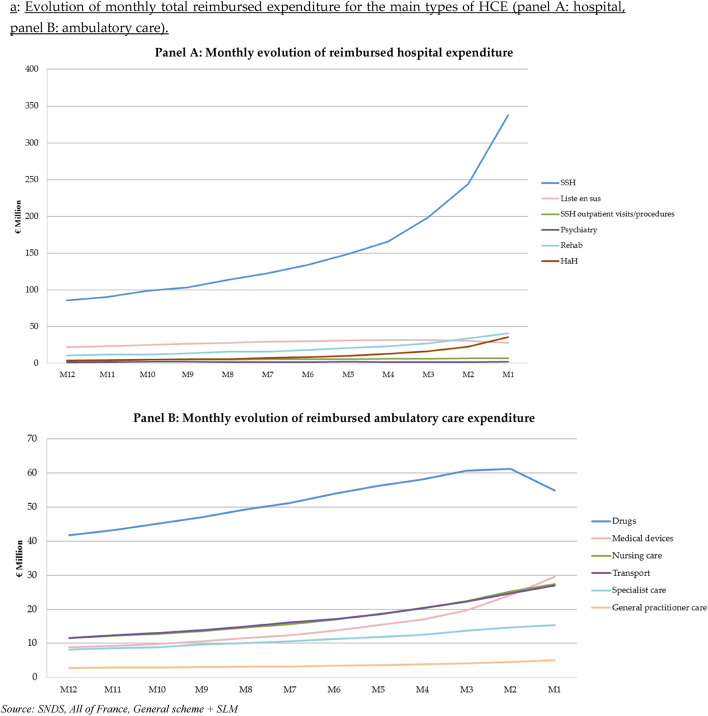

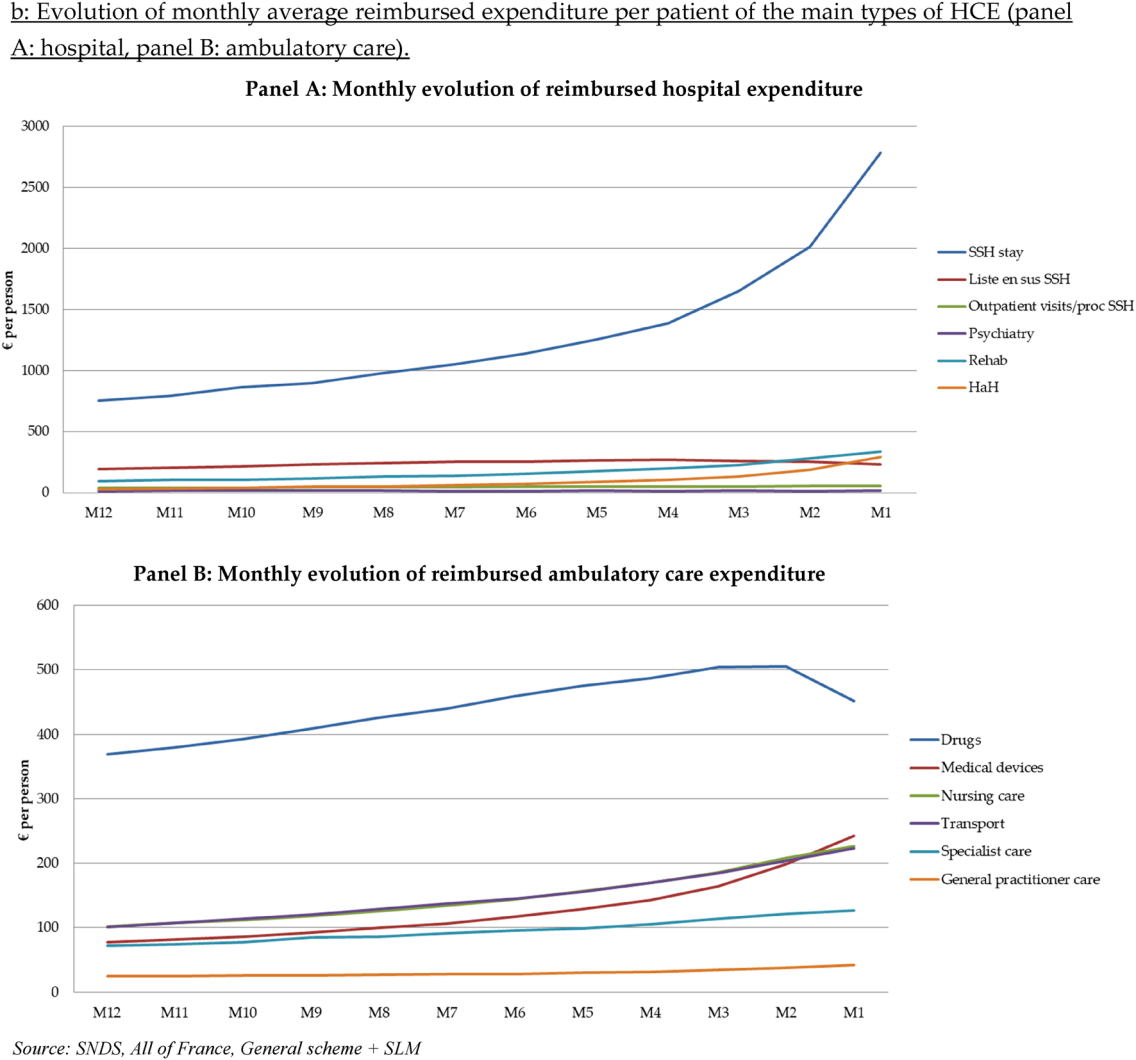
**a** Evolution of monthly total reimbursed expenditure for the main types of HCE (panel A: hospital, panel B: ambulatory care). Source: SNDS, all of France, General scheme + SLM. **b** Evolution of monthly average reimbursed expenditure per patient of the main types of HCE

Total reimbursed expenditure for all people who died in 2015 with an active cancer was close to €4.3 billion (including the month of death, but which was not recalculated) (Supplementary Table S1). About 62% of this expenditure is for hospital care, 35% for ambulatory care and 3% for cash benefits. Expenditure for hospital care and ambulatory care changed dramatically over the last 12 months of life (Fig. 1-Panels A and B). First, SSH expenditure increased relatively linearly between the 12th month (M12) and the 4th month (M4) before death. Since M4, SSH expenditure increased dramatically, especially the last month before death. "HaH" expenditure also increased tremendously between M2 and M1. Drugs represented the highest item among ambulatory care expenditure, although drug expenditure decreased sharply between M2 and M1. Marked growth of medical device expenditure was observed throughout the year, especially the last 3 months before death. This expenditure item became the second leading expenditure at M1.

Proportion of patients who used services (“utilization rate”, hereafter) increased progressively during the last months of life, regardless expenditure items (Table 2). Twelve months before the month of death (M12), 90% of people had, at least, one health care utilization and this proportion increased to 97% during M1 (Table 2). This growth of health care utilization rate was mainly related to an increase in hospital stays (increasing from 39% at M12 to 71% at M1), particularly SSH stays (increasing from 26% at M12 to 56% at M1). Ambulatory care utilization rate also increased, but less than for hospital care. Ambulatory care utilization increased from 89% at M12 to 93% at M1, i.e. + 4 percentage points (pp) versus + 33 pp for hospital care utilization rate. The highest growth rates were observed for transport and medical devices expenditure, which increased from 27% at M12 to 62% at M1 for "transport" and from 34% at M12 to 55% at M1 for "medical devices".

**Table 2:** Evolution of the monthly proportion of individuals using each type of health care expenditure item during the year before death (% of patients, *n* = 125,497)

	M12	M11	M10	M9	M8	M7	M6	M5	M4	M3	M2	M1	M0
	%	%	%	%	%	%	%	%	%	%	%	%	%
Total reimbursed expenditure	90.3	90.8	91.4	91.9	92.5	93.0	93.8	94.5	95.1	95.9	96.6	96.7	79.0
Total hospital expenditure	38.7	40.8	42.5	44.5	46.6	48.9	51.0	53.7	56.6	60.4	65.1	71.3	71.5
SSH stay	25.6	27.2	28.9	30.5	32.7	34.6	36.6	39.1	41.7	45.3	49.8	56.3	65.8
“Liste en sus” SSH	7.0	7.4	7.9	8.4	8.8	9.2	9.5	9.8	10.1	10.0	9.7	9.2	7.4
Outpatient visits/proc SSH	22.2	23.5	24.1	25.2	26.0	27.0	28.0	29.0	30.3	31.5	32.7	33.1	22.4
Psychiatry	0.1	0.2	0.1	0.2	0.1	0.1	0.2	0.2	0.2	0.2	0.1	0.2	0.1
Rehab	1.7	1.8	1.8	2.0	2.2	2.4	2.6	3.1	3.5	4.1	5.1	6.6	9.1
HaH	0.5	0.6	0.7	0.8	0.9	1.0	1.2	1.4	1.7	2.3	3.2	5.3	9.2
Total ambulatory care expenditure *including*	89.3	89.8	90.4	90.9	91.4	91.9	92.6	93.3	93.7	94.3	94.5	93.2	72.6
General practitioner care	53.0	53.7	54.4	54.9	55.6	56.5	57.2	58.1	59.2	60.5	61.2	58.7	37.5
Specialist care	29.9	30.2	30.9	31.5	32.0	32.3	32.9	33.5	34.2	34.6	34.4	31.4	13.8
Dental care	5.1	5.1	4.9	4.7	4.7	4.5	4.5	4.2	3.9	3.4	2.8	1.9	0.5
Physiotherapy	12.9	13.0	13.2	13.5	14.0	14.4	14.7	15.3	15.8	16.2	16.6	16.3	9.7
Nursing care	40.1	41.3	42.7	44.0	45.5	47.1	48.6	50.1	51.6	52.9	53.4	49.5	27.5
Laboratory tests	45.5	46.8	48.0	49.5	50.9	52.3	53.7	55.0	56.6	57.6	57.5	52.2	23.6
Drugs	82.3	82.8	83.3	83.8	84.2	84.6	84.9	85.3	85.0	84.4	82.3	74.5	40.4
Medical devices	34.4	35.5	36.6	37.9	39.3	41.0	42.7	44.7	46.9	49.6	53.0	54.9	36.4
Transport	26.7	28.3	29.7	31.5	33.4	35.6	37.8	40.4	43.7	47.7	53.5	61.7	43.0
Total cash benefits *including*	8.2	8.4	8.6	8.8	9.1	9.4	9.6	9.9	10.2	10.5	10.8	11.0	9.4
Sickness benefits	4.9	5.1	5.3	5.5	5.7	5.9	6.2	6.4	6.7	6.9	7.2	7.4	5.5
Disability benefits	3.5	3.5	3.6	3.6	3.6	3.7	3.7	3.8	3.8	3.9	3.9	3.9	4.1

### Monthly evolution of average reimbursed expenditure during the last 12 months of life

The last year of life average reimbursed expenditure (excluding M0 expenditure computation) was €34,273 per patient (Table 3). The average monthly expenditure increased progressively with the PTD from €2021 (M12) to €5207 (M1). Extrapolation of the average reimbursed expenditure during the month of the death (M0) was about €24,700 per patient. Hospital care was the main expenditure item but also the main driver of the increase: hospital care represented 56% of M12 expenditure but 71% of M1 expenditure and 93% of M0 one. Among hospital care expenditure, the highest average expenditure was observed for "SSH stays" with an average expenditure increasing from €756 (M12) to €2782 (M1) (+ 268%). A sharp growth of average expenditure was also observed for "Rehab" and "HaH".

**Table 3:** Average reimbursed expenditure per patient and per month during the year before death (€)

	M12	M11	M10	M9	M8	M7	M6	M5	M4	M3	M2	M1	M0	Total
Average reimbursed expenditure	2021	2122	2244	2363	2523	2664	2843	3068	3311	3713	4267	5207	24,766	34,273
Average hospital expenditure	1127	1196	1283	1354	1467	1560	1679	1846	2022	2340	2802	3714	22,933	21,147
SSH stay	756	793	863	897	981	1049	1137	1253	1387	1650	2013	2782	18,617	14,709
*Liste en sus* SSH	196	206	217	231	242	252	254	263	268	262	255	230	750	2698
Outpatient visits/proc SSH	37	40	42	43	45	46	49	50	51	52	54	53	131	528
Psychiatry	11	15	17	18	16	14	14	18	13	16	14	19	75	173
Rehab	96	106	103	118	135	136	152	177	197	225	280	337	1589	1949
HaH	31	35	42	48	50	62	73	86	107	134	186	292	1771	1091
Average ambulatory care expenditure *including*	804	833	866	910	955	1000	1056	1113	1176	1258	1347	1380	1567	11,945
General practitioners	25	25	26	26	27	28	28	30	32	34	37	42	82	338
Specialists	72	75	78	84	86	91	96	99	105	114	121	127	114	1080
Dentists	4	4	4	3	3	3	3	3	3	2	2	1	1	34
Physiotherapists	21	21	22	22	23	24	25	25	26	27	28	27	24	273
Nurses	102	107	112	118	126	134	144	157	169	186	208	226	255	1687
Laboratory tests	29	30	31	32	34	35	36	38	39	40	40	37	28	396
Drugs	369	380	393	409	426	439	459	475	486	504	505	452	358	4972
Medical devices	78	81	85	92	99	106	117	129	142	164	198	243	422	1449
Transport	101	107	114	121	128	138	145	155	170	185	204	223	250	1688
Average cash benefits	91	93	95	99	100	104	108	108	114	115	118	113	267	1181
Sickness benefits	57	59	60	63	65	68	72	72	76	78	80	75	68	776
Disability benefits	34	34	35	35	35	35	36	36	37	37	38	38	198	405

Yearly average ambulatory care expenditure was lower than average hospital expenditure (€11,945 versus €21,147). “Drugs” (prescribed in pharmacies) was the item with the highest average expenditure (about €5000 per patient), monthly expenditure increasing from €369 per patient at M12 to €505 per patient at M2 with a decrease the following months. “Nursing care” and “transport” displayed expenditure of about €1700 per patient over the 12 months before the month of death. The preponderance of these two expenditure items in the overall average ambulatory expenditure remains relatively constant throughout the last year of life. It is not the case for “medical devices” (€1449 per patient over the period) with a monthly average expenditure which increased sharply over the period: €78 for M12 to €243 for M1 and €422 for M0.

### Average reimbursed expenditure according to the patient's age and the quarter considered

Average reimbursed expenditure during the twelve months preceding the month of death tended to decrease as people's age increases (Table 4). Average reimbursed expenditure was slightly more than €50,000 per patient aged 18–59 and less than €18,000 per patient for people 90 + . Regardless of age, about 60% of HCE involved hospital care even though this expenditure decreased markedly with increasing age. Average hospital expenditure for people 18–59 was close to €30,000 versus €10,600 for patients 90 + . In particular, expenditure related to drugs and device out of DRG system (*“liste en sus”*) decreased by more than 90% (from €5145 to €360, per patient). Only "Rehab" item increased with age, from €1496 for 18–59 years versus €2267 for people 90 + , per patient. This increase can also be explained by a much higher utilization rate as people’s age increases (12.5% versus 23%, Table S2). Ambulatory care expenditure growth rate was similar to that observed for hospital care (€15,200 for 18–59 versus €7000 for people 90 +), but with more marked variations according to expenditure item considered. Average GP expenditure was higher for people 90 + than for people 18–59 years (€398 per patient versus €283 per patient), while average reimbursed specialist expenditure were highest for the youngest patients (€1471 per patient versus €356 per patient). Differences in average GP expenditure cannot be explained by differences in the proportion of individuals who have contact with GP (about 96.5% for both age groups) but could be due to a greater number of visits. Differences in specialist expenditure could be related to decreasing utilization rate of specialist care with increasing age (87% versus 75%, for 18–59 and 90 + age groups).

**Table 4:** Quarterly average reimbursed expenditure per patient and per month during the year before death (€)

	18–59 years					60–70 years					70–80 years					80–90 years					≥ 90 years				
	Q4	Q3	Q2	Q1	Total	Q4	Q3	Q2	Q1	Total	Q4	Q3	Q2	Q1	Total	Q4	Q3	Q2	Q1	Total	Q4	Q3	Q2	Q1	Total
Average reimbursed expenditure	9386	11,222	13,705	18,249	50,342	7186	8581	10,414	14,740	39,699	6001	7254	8979	13,017	34,676	4488	5165	6407	9856	25,623	3102	3584	4411	6653	17,504
Average hospital expenditure	5126	6311	7962	11,525	29,676	4195	5112	6301	9890	24,768	3484	4328	5531	8927	21,923	2493	2954	3884	6873	16,029	1590	1940	2626	4563	10,575
SSH	3336	4059	5247	8180	20,007	2772	3426	4305	7222	17,234	2360	2938	3808	6598	15,465	1710	2005	2706	5042	11,342	1093	1317	1767	3256	7334
*Liste en sus*	1125	1380	1503	1397	5145	842	972	1054	980	3715	597	681	760	726	2713	266	305	313	321	1189	67	90	93	115	360
SSH outpatient visits/procedure	191	215	236	237	836	150	169	190	200	685	116	135	147	161	548	67	75	86	98	321	34	38	42	50	161
Psychiatry	106	110	111	105	410	65	52	53	74	235	28	43	36	29	133	12	19	12	19	62	6	8	8	19	40
Rehab	210	301	429	614	1496	242	318	410	699	1624	275	367	492	824	1929	360	443	612	1005	2394	360	427	617	895	2267
HaH	159	246	437	992	1782	125	173	289	715	1276	108	164	289	589	1135	77	107	155	390	722	31	61	98	227	412
Average ambulatory care expenditure *including*	3000	3525	4217	5111	15,156	2682	3156	3797	4543	13,730	2514	2921	3442	4085	12,735	1994	2211	2523	2983	9593	1512	1644	1785	2090	6929
General practitioners	62	67	75	92	283	63	69	78	98	299	70	76	87	111	338	80	86	97	125	382	86	90	100	127	398
Specialists	299	352	400	488	1471	284	329	385	475	1426	225	270	312	374	1161	148	171	196	232	738	78	85	96	101	356
Nurses	238	313	438	645	1572	247	314	429	600	1544	278	341	443	610	1645	367	410	477	584	1817	442	475	509	561	1957
Drugs	1527	1724	1972	2074	6955	1282	1480	1698	1758	6009	1206	1357	1508	1530	5497	814	875	953	958	3553	437	464	470	506	1849
Medical devices	272	356	492	812	1861	273	338	453	693	1708	241	299	388	608	1511	199	228	287	440	1141	156	178	216	317	855
Transport	447	538	646	795	2321	377	455	562	721	2050	328	397	495	642	1830	220	261	314	440	1222	128	154	184	259	715

Source: SNDS, All of France, General scheme + SLM

Quarterly analysis showed that average reimbursed expenditure tended to increase with the PTD, with the highest expenditure growths observed for SSH, Rehab and HaH. These high expenditure growth rates reflect, among other things, the marked increase in the proportion of patients using this service (Table S2).

### Out-of-pocket payments

Consistent with previous results in terms of average reimbursed expenditure, average out-of-pocket (OOP) increased (Table 5), especially with the PTD. For example, average OOP (all expenditure items considered) for people 60–69 years increased from €300 (Q4) to €583 (Q1). This OOP growth was essentially due to hospital expenditure, for which OOP increased with time, regardless of age group. Nevertheless, the great majority of OOP’s growth rates were lower than those for expenditure.

**Table 5:** Quarterly average out-of-pocket payments per patient using the type of care during the year before death according to age (€)

	18–59 years					60–69 years					70–79 years					80–89 years					≥ 90 years				
	Q4	Q3	Q2	Q1	Total	Q4	Q3	Q2	Q1	Total	Q4	Q3	Q2	Q1	Total	Q4	Q3	Q2	Q1	Total	Q4	Q3	Q2	Q1	Total
Average reimbursed expenditure	307	356	416	604	1611	300	334	384	583	1553	305	335	398	591	1602	315	341	401	591	1628	317	338	393	528	1552
Average hospital expenditure	222	246	276	436	1038	210	228	257	420	964	213	226	272	428	970	243	263	299	449	965	286	305	344	435	885
SSH	221	245	275	435	882	210	228	257	419	805	213	226	272	428	783	243	263	299	449	738	286	305	344	435	661
SSH outpatient visits/procedures	11	11	10	10	30	9	9	9	9	24	8	8	8	9	22	9	10	10	10	22	12	12	11	13	23
Psychiatry	766	780	677	793	1118	865	791	554	946	1174	891	959	1120	704	1345	636	589	483	434	671	236	491	778	881	742
Rehab	715	760	789	706	1032	758	736	752	689	992	687	770	757	715	997	750	740	790	723	1007	794	810	842	743	1047
HaH	28	36	26	23	37	8	9	16	21	24	13	5	19	27	30	14	33	27	30	39	26	48	104	72	97
Average ambulatory care expenditure *including*	154	162	165	163	607	162	164	166	160	626	175	173	174	164	671	186	186	188	180	728	199	197	196	198	774
General practitioners	11	11	10	9	33	9	9	9	9	30	9	9	9	10	31	10	10	10	12	38	13	13	14	15	49
Specialists	34	32	28	23	76	33	30	29	27	80	37	35	33	30	94	38	36	36	31	91	38	39	36	31	82
Nurses	9	10	13	12	32	10	10	11	11	30	10	10	11	10	31	19	18	19	19	56	41	42	43	43	125
Drugs	45	49	54	57	187	48	49	51	54	189	51	52	53	54	214	55	55	56	55	217	56	56	56	57	222
Medical devices	87	86	79	67	209	87	85	77	63	213	91	83	76	61	218	95	91	83	73	238	99	91	88	77	239
Transport	16	15	16	18	46	13	14	15	18	41	13	13	14	17	38	14	15	15	20	41	19	21	22	25	50

Furthermore, OOP did not decrease with increasing age, as the average total OOP was €1553 for the 60–69 years age group and €1552 for people 90 + . Consequently, the OOP for the youngest people (who had the highest average expenditure, Table 4) were not much higher than that of the people with the lowest average expenditure. 18–59-year-old individuals had an average expenditure 2.9-fold higher than that of people 90 + , but the OOP ratio was only 1.04. Furthermore, hospital OOP tended to decrease with age, decreasing from €1038 for the 18–59 years age group to €885 for people 90 + . This declining trend of hospital OOP was parallel to that of total expenditure (Table 4). Inversely, ambulatory care OOP increased with age, in contrast with total expenditure: increasing from €607 per patient for the 18–59 years age group to €774 per patient for people 90 years and older.

Analysis of hospital OOP by expenditure item and quarter showed that OOP related to SSH hospitalization varied only slightly during the first three quarters, but then increased considerably during the last quarter. For example, the OOP of people between the ages of 70 and 79 years with at least one SSH stay increased from €213 to €272 during the first three quarters to reach €428 during the last quarter. This increase in OOP during the last quarter can be explained by the marked increase in utilization rates during this quarter, regardless of age (Table S2). Ambulatory items with the highest OOP were: "medical devices", "drugs" and "specialists". OOP related to nursing care were particularly high for people 90 years and older. OOP related to "specialists" and "medical devices" decreased throughout the year although, average expenditure increased for each quarter (Table 4). Average OOP related to "drugs" increased during the last quarter for people between the ages of 18 and 79 years, while remained stable throughout the year for people 80 years and older.

## Discussion and conclusion

This study, conducted on SNDS data on 125,000 people treated for cancer and died in 2015, provides detailed information on monthly reimbursed HCE and OOP at the end of life. One of the main strengths of this study is the utilization of SNDS data, ensuring comprehensive data on ambulatory, hospital expenditure and cash benefits [32]. It is thus possible to conduct more detailed analysis in terms of expenditure items where most studies in the literature focus mainly on one care item (drugs, hospitalization, etc.) [1, 2, 12, 34]. Moreover, this database encompasses nearly 80% of the French population. Another strength using SNDS database is that it allows for a long-term follow-up. In addition, the use of medical administrative data considerably limits the risk of memory bias concerning both HCE and OOP. This bias is particularly prevalent in OOP studies because of the frequent use of survey data in this field [8, 26–28].

The results of our study were consistent with those in the literature. First, average reimbursed expenditure over the last 12 months before the month of death in 2015 for people with a cancer was about €34,300 which was higher than for all French population combined, for which the average expenditure was €17,000 [19]. However, many studies had shown that end-of-life expenditure of people with cancer are higher than that of people with other diseases [14–16, 35]. Average expenditure may also vary according to the cancer type. Several studies based on the same population and methodology reported specific average expenditure during the last year of life (colorectal: €43,400, lung: €43,300, prostate: €38,750, breast: €45,418) [29, 36, 37]. Our global cancers’ HCE are lower than those for specific cancer. In fact, this result is due to the case-mix of cancers in our study population. 40% of this population is composed of people with cancers with higher expenditure (mainly lung, breast and colorectal cancer) than the average expenditure of people with active cancer.^4^ As a consequence, our average expenditure for all cancers are thus lower than those presented in the others studies.

Second, expenditure increased with PTD, regardless of age, progressively rising from an average monthly expenditure of €2000 (M12) to €5200 during the last month before the month of death (M1). This growth of expenditure with the PTD is consistent with the results of the "red herring" literature, which concluded that the high level of HCE was due more to the PTD than to the individual's age per se [6–8]. Third, average end-of-life HCE were lower with increasing age from €50,300 for people 18–59 years old to €17,500 for people 90 +. This result is consistent with several studies conducted in different contexts: England [6], USA [38], The Netherlands [18] and Korea [35]. Finally, HCE towards the end-of-life in France were mainly hospital expenditure. This predominant role of hospital care is also consistent with the results of other studies [22, 24, 25].

In addition to these results, our study provides new insights into the analysis of end-of-life expenditure.

First, medical devices and related services expenditure increased progressively with the PTD. This increase can be the sign of greater use of home support for people at the end of life. Based on the same population, a study pointed out the high utilization rate of medical devices and related services by people predominantly managed at home during their last month of life in 2015 [29]. This increase in medical devices and related services utilization can also reflect patients’ preference for death at home [22]. However, as mentioned by Tuppin et al., “among the 20% of cancer patients treated mainly at home during their last month of life in 2015, almost one-half finally died in hospital shortly after admission”[29]. These results therefore raised the question of the place of palliative care in the end-of-life management of people with cancer, especially as the use of palliative or supportive care in this setting allowed an improvement of the patient's quality of life [39–41].

Second, our results showed that average out-of-pocket payments for people with cancer at the end of life represented between 3 and 9% of average reimbursed expenditure according to expenditure item. The percentage of out-of-pocket payments increased with age, but the actual sums remained very similar, as the average OOP was €1611 for people 18–59 years old vs. €1552 for people 90 + . Thus, even with a growing amount of HCE, patients faced similar amount of OOP. In France, for most expenditure items (e.g. drugs, biology, most of GP…) OOP consist only of co-payments due to an administered price system. In the same time, individuals with particular expensive pathologies (e.g. cancer, HIV, multiple sclerosis…) can be covered by the long-term disease (LTD) scheme which provides 100% coverage of co-payments related to the pathology. A great part of patients with cancers are LTD scheme’s beneficiaries and thus are covered for cancer-linked co-payments. Even if patients are not LTD scheme’s beneficiaries, they can have a complementary health insurance (CHI) which can also covered co-payments. Contrary to LTD beneficiaries, CHI covered patients had to pay an insurance premium. Another part of OOP is extra-billing for health professionals and free selling prices in excess of the statutory tariff. This kind of OOP is not covered by LTD and can be partly or entirely covered by certain CHI, depending of the insurance contract. OOP analyzed in this study are those before CHI intervention and thus are probably over estimated for the great majority of the patients. Despite the LTD scheme and the possibility to have a CHI, very high concentration of OOP are likely to occur [42]. However, thanks to all these schemes, France has one of the lowest levels of out-of-pocket payments of all OECD countries, accounting for about 10% of total HCE.^5^ France is therefore the country with the second lowest percentage of OOP after South Africa (7.7% in 2015), with a much lower rate than in countries such as Australia (19%) or Korea (34%).

Our comprehensive data allow analyzing a specific item expenditure which is drugs and medical devices out DRG tariffs that promotes access to innovative devices. Results clearly indicate an item’s expenditure growth in PTD but average expenditure was lower as patients’ age increased. This important difference may be the consequence, on one hand, of marked disparities in the utilization rate of innovative drugs and medical devices according to age. Indeed, the utilization rate for this expenditure item is 10.2% for people 90 + versus more than 45% for 18–59 years (Table S2). These results may raise the question of equal access to innovative drugs and medical devices even in a fully reimbursement scheme. A recent study analyzed all incident cases of metastatic lung cancer hospitalized for a chemotherapy in public hospitals in 2011 and their access to innovative drugs [43]. They showed that the probability of prescription of innovative drugs is inversely related to age. Similar results are also found in different contexts [44]. On the other hand, older individuals may have more comorbidities or be diagnosed later, because they are no longer in the organized screening age groups, which may contraindicate the use of these innovative drugs.

This study has several limitations. First, this study was based on administrative reimbursement data; coding errors are therefore always possible. Furthermore, data are only available for general scheme beneficiaries and for reimbursements for people treated for their cancer. Their causes of death are in the process of being included in the SNDS and could be linked for 94% of them. Based on the same population, 81% of patients had a tumor as the main cause of death [45]. However, as we did not focus on cancer-specific HCE, there may be no concern about global HCE for the rest of the population. Failure to take into account nursing home expenditure may have artificially accentuated the decline in expenditure over the 12 months before the death of the oldest people, as the proportion of institutionalized people increased with age. However, as only a small proportion of people, about 5%, were institutionalized, nursing home expenditure would consequently not have been sufficient to reverse the overall trend [46]. In 2015, the general scheme covered 77% of the French population. The rest is covered by other compulsory health insurance schemes (mainly farmers, self-employed, civil servants or students) and was beyond the scope of this study. No information concerning private health insurance was available. Consequently, the OOP presented here may have been partially or fully reimbursed by private health insurance, which would further reduce the real OOP. Our dataset did not encompass expenditure items that are not covered by the general scheme. As a result, a part of the actual total expenses financed by CHI or directly by individuals are not available in our data. The M0 may be overestimated if people presented on only 1 day in the month had particularly large expenses on that day alone. Nevertheless, the same applied for individuals with very low expenses on that day. Moreover, individuals in the last month are presented in average 14 days. We can thus assume a two-fold overestimation.

Despite these limitations, our study provides interesting information for decision makers. In particular, the French LTD system allows a 100% coverage of cancer-related HCE and thus limits the amount of OOP. This scheme ensures a very good coverage of expenses directly related to cancer. Moreover, OOP can be partially or completely covered, depending of the CHI’s contract. Despite this system, individual disparities may exist, with possible dramatic OOP for some person, which should be studied more specifically. In the same way, further studies on disparities in the use of innovations and the place/impact of palliative care near to death for people with cancer would be interesting in order to help policy maker to provide a more efficient access to this type of care.

## Supplementary Information

Below is the link to the electronic supplementary material.

Supplementary file1 (DOCX 30 KB)

## Footnotes

1 Sum converted with the January 2014 exchange rate and considering inflation up until December 2015 to be consistent with the data presented in this study.

2 https://www.ameli.fr/l-assurance-maladie/statistiques-et-publications/etudes-en-sante-publique/cartographie-des-pathologies-et-des-depenses/index.php

3 Medical devices encompass wheelchairs, medical beds or prostheses for example. Related services are mainly rental packages such as oxygen therapy, insulin pumps, etc.

4 https://www.ameli.fr/l-assurance-maladie/statistiques-et-publications/etudes-en-sante-publique/cartographie-des-pathologies-et-des-depenses/depenses-remboursees-affectees-a-chaque-pathologie.php

5 https://data.oecd.org/healthres/health-spending.htm
