# Supplementary material for: Evolution of health care utilization and expenditure during the year before death in 2015 among people with cancer: French snds-based cohort study
Source: Eur J Health Econ. 2021 Jun 7;22(7):1039–52. doi: 10.1007/s10198-021-01304-1 (PMC8318964; doi:10.1007/s10198-021-01304-1)
Supplement: Supplementary file 1 — Supplementary file1 (DOCX 30 KB) [file 10198_2021_1304_MOESM1_ESM.docx]

Supplementary tables

Table S1: Evolution of total reimbursed HCE during the year before death (€ millions)

|  | **M12** | **M11** | **M10** | **M9** | **M8** | **M7** | **M6** | **M5** | **M4** | **M3** | **M2** | **M1** | **M0** | **Total** |
| --- | --- | --- | --- | --- | --- | --- | --- | --- | --- | --- | --- | --- | --- | --- |
| **Total reimbursed expenditure** | **229.1** | **241.9** | **257.5** | **272.6** | **292.8** | **311.0** | **334.5** | **363.8** | **395.5** | **447.1** | **517.5** | **632.1** | **2 458.7** | **4 295.3** |
| **Total hospital expenditure** | 127.7 | 136.3 | 147.2 | 156.2 | 170.3 | 182.2 | 197.5 | 218.9 | 241.6 | 281.8 | 339.8 | 450.9 | **2 276.7** | 2 650.3 |
| SSH stay | 85.7 | 90.4 | 99.0 | 103.4 | 113.8 | 122.5 | 133.8 | 148.5 | 165.7 | 198.7 | 244.1 | 337.7 | 1 848.2 | 1 843.4 |
| SSH *Liste en sus* | 22.2 | 23.4 | 24.9 | 26.6 | 28.0 | 29.4 | 29.9 | 31.2 | 32 | 31.5 | 30.9 | 28 | 74.5 | 338.1 |
| SSH outpatient visits/procedures | 4.2 | 4.6 | 4.8 | 5.0 | 5.2 | 5.4 | 5.7 | 5.9 | 6.1 | 6.3 | 6.5 | 6.5 | 13.0 | 66.1 |
| Psychiatry | 1.3 | 1.8 | 2.0 | 2.0 | 1.8 | 1.6 | 1.6 | 2.1 | 1.5 | 1.9 | 1.7 | 2.3 | 7.5 | 21.7 |
| Rehab | 10.9 | 12.1 | 11.8 | 13.6 | 15.6 | 15.9 | 17.9 | 21.0 | 23.5 | 27.1 | 34.0 | 40.8 | 157.8 | 244.2 |
| HaH | 3.5 | 4.0 | 4.8 | 5.5 | 5.8 | 7.2 | 8.6 | 10.2 | 12.8 | 16.2 | 22.5 | 35.6 | 175.8 | 136.7 |
| **Total ambulatory care expenditure** *including* | **91.1** | **95.0** | **99.3** | **105.0** | **110.8** | **116.7** | **124.3** | **132.0** | **140.4** | **151.4** | **163.4** | **167.4** | **155.5** | **1 497.0** |
| General practice care | 2.8 | 2.9 | 2.9 | 3.0 | 3.1 | 3.2 | 3.4 | 3.5 | 3.8 | 4.1 | 4.5 | 5.1 | 8.1 | 42.3 |
| Specialist care | 8.2 | 8.5 | 8.9 | 9.7 | 10.0 | 10.6 | 11.3 | 11.8 | 12.5 | 13.7 | 14.7 | 15.4 | 11.3 | 135.3 |
| Dental care | 0.4 | 0.4 | 0.4 | 0.4 | 0.4 | 0.4 | 0.4 | 0.4 | 0.3 | 0.3 | 0.2 | 0.2 | 0.1 | 4.2 |
| Physiotherapy | 2.4 | 2.4 | 2.5 | 2.6 | 2.7 | 2.8 | 2.9 | 3.0 | 3.2 | 3.3 | 3.4 | 3.2 | 2.4 | 34.2 |
| Nursing care | 11.6 | 12.2 | 12.8 | 13.6 | 14.6 | 15.6 | 16.9 | 18.6 | 20.2 | 22.4 | 25.2 | 27.4 | 25.3 | 211.4 |
| Laboratory tests | 3.3 | 3.4 | 3.6 | 3.7 | 3.9 | 4.1 | 4.3 | 4.5 | 4.7 | 4.8 | 4.9 | 4.4 | 2.8 | 49.6 |
| Drugs | 41.8 | 43.3 | 45.1 | 47.1 | 49.4 | 51.3 | 54.0 | 56.3 | 58.1 | 60.7 | 61.3 | 54.9 | 35.5 | 623.2 |
| Medical devices | 8.8 | 9.3 | 9.8 | 10.6 | 11.5 | 12.4 | 13.7 | 15.3 | 17 | 19.7 | 24.1 | 29.5 | 41.9 | 181.7 |
| Transport | 11.5 | 12.3 | 13.1 | 13.9 | 14.9 | 16.1 | 17.1 | 18.4 | 20.3 | 22.2 | 24.7 | 27 | 24.9 | 211.5 |
| **Total cash benefits** | **10.3** | **10.6** | **10.9** | **11.4** | **11.7** | **12.1** | **12.7** | **12.9** | **13.5** | **13.9** | **14.3** | **13.8** | **26.5** | **148.1** |
| Sickness benefits | 6.4 | 6.7 | 6.9 | 7.3 | 7.6 | 8.0 | 8.4 | 8.6 | 9.1 | 9.4 | 9.7 | 9.1 | 6.8 | 97.2 |
| Disability benefits | 3.8 | 3.9 | 4.0 | 4.1 | 4.0 | 4.1 | 4.2 | 4.3 | 4.4 | 4.5 | 4.6 | 4.7 | 19.7 | 50.7 |

*Source: SNDS, All of France, General scheme + SLM*

Table S2: Evolution of the quarterly proportion of individuals using each type of health care expenditure item during the year before death (% of patients, n = 125,497)

|  | **18 – 59 years**  **(n= 20,574)** | | | | | **60 – 69 years**  **(n= 28,743)** | | | | | **70 – 79 years**  **(n= 29,719)** | | | | | **80 – 89 years**  **(n= 34,678)** | | | | | **≥ 90 years**  **(n= 11,783)** | | | | |
| --- | --- | --- | --- | --- | --- | --- | --- | --- | --- | --- | --- | --- | --- | --- | --- | --- | --- | --- | --- | --- | --- | --- | --- | --- | --- |
|  | **Q4** | **Q3** | **Q2** | **Q1** | **Total** | **Q4** | **Q3** | **Q2** | **Q1** | **Total** | **Q4** | **Q3** | **Q2** | **Q1** | **Total** | **Q4** | **Q3** | **Q2** | **Q1** | **Total** | **Q4** | **Q3** | **Q2** | **Q1** | **Total** |
| **Average reimbursed expenditure** | **90.2** | **93.0** | **96.1** | **99.3** | **99.7** | **93.5** | **95.0** | **96.8** | **99.4** | **99.8** | **96.8** | **97.3** | **98.3** | **99.5** | **99.9** | **98.1** | **98.3** | **98.7** | **99.5** | **99.9** | **98.1** | **98.2** | **98.5** | **99.0** | **99.9** |
| **Average hospital expenditure** | **65.0** | **72.8** | **81.8** | **93.1** | **96.6** | **62.8** | **69.9** | **78.3** | **91.7** | **96.0** | **61.9** | **68.2** | **75.7** | **89.6** | **96.0** | **53.1** | **57.7** | **64.9** | **80.8** | **93.3** | **41.3** | **44.9** | **51.0** | **67.2** | **87.9** |
| SSH | 50.4 | 59.5 | 70.5 | 85.2 | 93.3 | 47.5 | 55.9 | 65.2 | 82.6 | 92.1 | 44.8 | 52.0 | 61.1 | 78.9 | 91.4 | 35.2 | 39.8 | 47.6 | 67.5 | 86.1 | 25.8 | 29.3 | 35.7 | 53.2 | 78.2 |
| *Liste en sus* | 17.6 | 22.0 | 25.6 | 27.8 | 45.3 | 14.9 | 17.5 | 20.1 | 22.2 | 38.3 | 11.8 | 13.7 | 15.6 | 17.7 | 31.8 | 6.5 | 7.1 | 8.1 | 9.7 | 19.5 | 2.4 | 2.7 | 3.2 | 4.7 | 10.2 |
| SSH outpatient visits/procedures | 51.2 | 57.3 | 63.7 | 70.0 | 86.1 | 47.6 | 52.7 | 58.7 | 65.8 | 82.3 | 45.5 | 50.2 | 54.3 | 60.6 | 79.6 | 36.4 | 39.2 | 43.2 | 48.4 | 72.0 | 25.5 | 27.0 | 29.5 | 34.1 | 60.3 |
| Psychiatry | 0.8 | 0.6 | 0.8 | 0.7 | 1.9 | 0.4 | 0.4 | 0.4 | 0.4 | 1.1 | 0.2 | 0.3 | 0.2 | 0.2 | 0.6 | 0.2 | 0.2 | 0.2 | 0.2 | 0.6 | 0.1 | 0.1 | 0.1 | 0.1 | 0.4 |
| Rehab | 2.2 | 3.2 | 4.5 | 7.6 | 12.5 | 2.9 | 3.7 | 4.9 | 9.2 | 15.1 | 3.7 | 4.5 | 6.3 | 11.2 | 18.8 | 4.9 | 5.8 | 8.0 | 13.8 | 24.1 | 5.0 | 5.6 | 7.9 | 12.0 | 23.0 |
| HaH | 1.4 | 2.2 | 3.9 | 10.6 | 12.5 | 1.1 | 1.6 | 2.9 | 7.9 | 9.6 | 1.1 | 1.6 | 2.6 | 6.8 | 8.4 | 0.7 | 1.0 | 1.5 | 4.6 | 5.7 | 0.4 | 0.6 | 1.0 | 2.7 | 3.5 |
| **Average ambulatory care expenditure** *including* | **89.5** | **92.3** | **95.5** | **98.7** | **99.5** | **93.1** | **94.7** | **96.5** | **99.0** | **99.7** | **96.6** | **97.1** | **98.1** | **99.2** | **99.9** | **97.9** | **98.0** | **98.4** | **99.0** | **99.9** | **97.8** | **97.8** | **98.0** | **98.4** | **99.9** |
| General practitioners | 72.8 | 76.1 | 78.5 | 80.2 | 96.4 | 78.3 | 80.2 | 82.3 | 83.6 | 97.4 | 84.8 | 85.4 | 86.2 | 86.2 | 98.1 | 88.1 | 88.3 | 88.7 | 87.5 | 98.0 | 86.9 | 86.6 | 86.1 | 85.4 | 96.5 |
| Specialists | 52.4 | 55.9 | 59.5 | 60.1 | 87.1 | 56.1 | 59.0 | 62.3 | 63.9 | 89.3 | 59.6 | 62.0 | 64.1 | 63.2 | 89.9 | 54.4 | 55.0 | 56.0 | 54.9 | 85.0 | 43.4 | 42.3 | 42.8 | 41.3 | 75.1 |
| Nurses | 49.4 | 56.9 | 64.5 | 70.4 | 84.9 | 54.1 | 60.2 | 66.9 | 73.4 | 86.9 | 58.7 | 63.2 | 68.5 | 72.9 | 88.0 | 60.0 | 62.4 | 65.5 | 67.9 | 85.0 | 53.3 | 54.1 | 54.8 | 55.4 | 73.7 |
| Drugs | 84.2 | 87.5 | 90.4 | 91.4 | 97.4 | 89.6 | 91.2 | 92.7 | 92.6 | 97.8 | 94.2 | 94.6 | 94.9 | 93.3 | 98.5 | 95.5 | 95.2 | 94.7 | 92.5 | 98.1 | 94.4 | 93.9 | 92.8 | 90.8 | 96.6 |
| Medical devices | 47.7 | 54.3 | 62.7 | 75.2 | 89.9 | 52.0 | 57.2 | 64.8 | 76.7 | 90.3 | 55.4 | 60.1 | 66.8 | 77.0 | 91.0 | 55.4 | 59.1 | 64.4 | 73.6 | 89.8 | 53.2 | 55.6 | 58.7 | 66.6 | 86.4 |
| Transport | 44.3 | 52.5 | 62.5 | 79.9 | 87.3 | 42.9 | 50.0 | 60.0 | 79.9 | 87.2 | 43.2 | 50.4 | 60.0 | 80.6 | 88.4 | 40.5 | 45.9 | 54.5 | 77.1 | 87.8 | 34.6 | 39.0 | 47.1 | 70.4 | 86.2 |

*Source: SNDS, All of France, General scheme + SLM*
